# Supplementary material for: A phenopushing platform to identify compounds that alleviate acute hypoxic stress by fast-tracking cellular adaptation
Source: Nat Commun. 2025 Mar 18;16:2684. doi: 10.1038/s41467-025-57754-1 (PMC11920246; doi:10.1038/s41467-025-57754-1)
Supplement: Supplementary file 1 — Supplementary Information [file 41467_2025_57754_MOESM1_ESM.pdf]

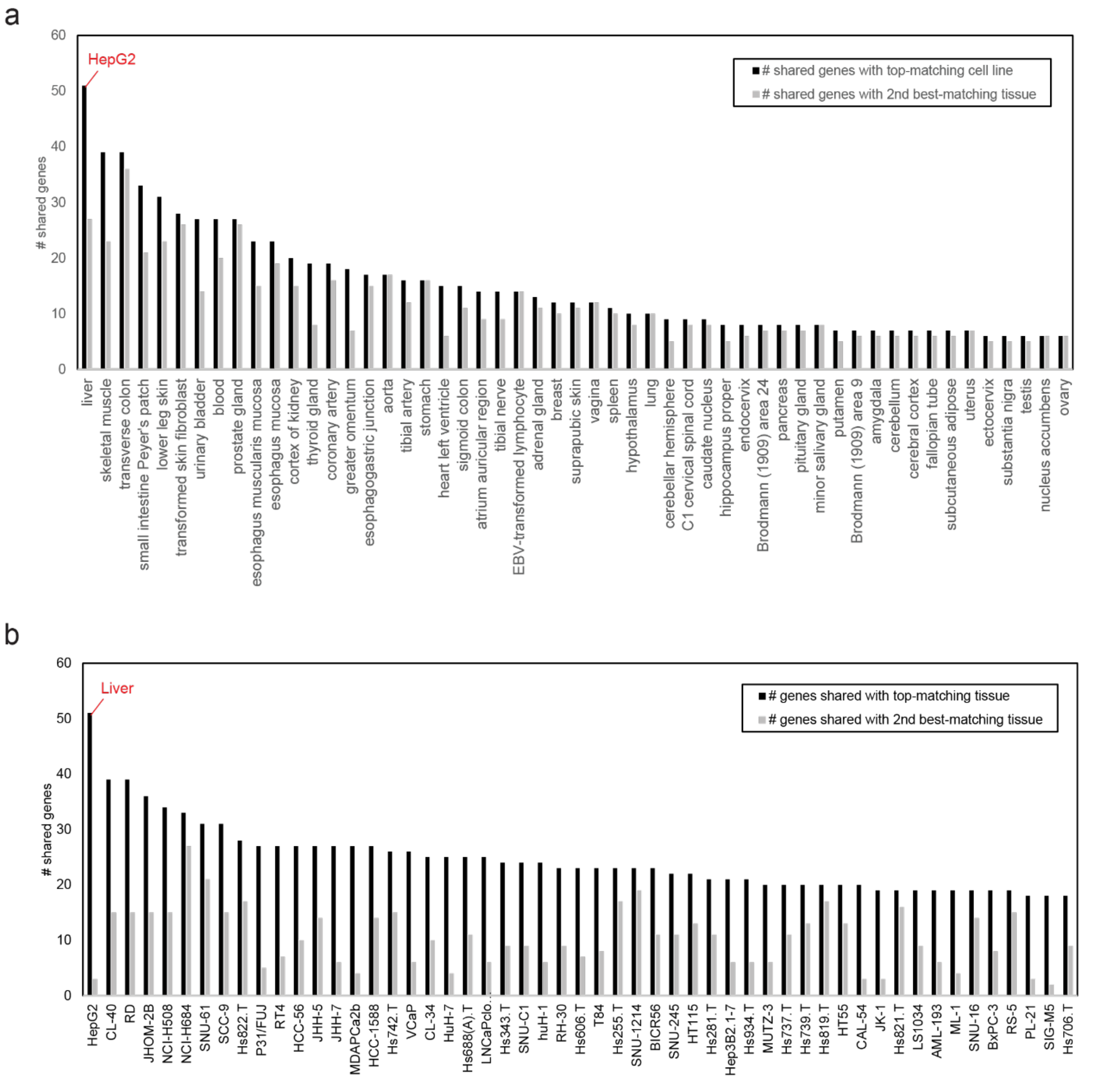

Supplementary Figure 1. Selection of HepG2 for phenotypic profiling of cellular response to hypoxia. To assess similarity between 934 human cell lines (CCLE and 53 human tissues), the top 200 most highly expressed genes were identified for each cell line and each tissue, and the number of genes shared in the "top 200 gene list" for pairwise cell line-tissue comparisons was determined. **(a)** For each tissue, the top two best-matching cell lines were identified. All 53 tissues are shown in the bar graph. **(b)** For each cell line, the top two best-matching tissues were identified. The top 53 cell lines (ranked by the number of genes shared with its top-matching tissue) are shown in the bar graph. Source data are provided as a Source Data file.

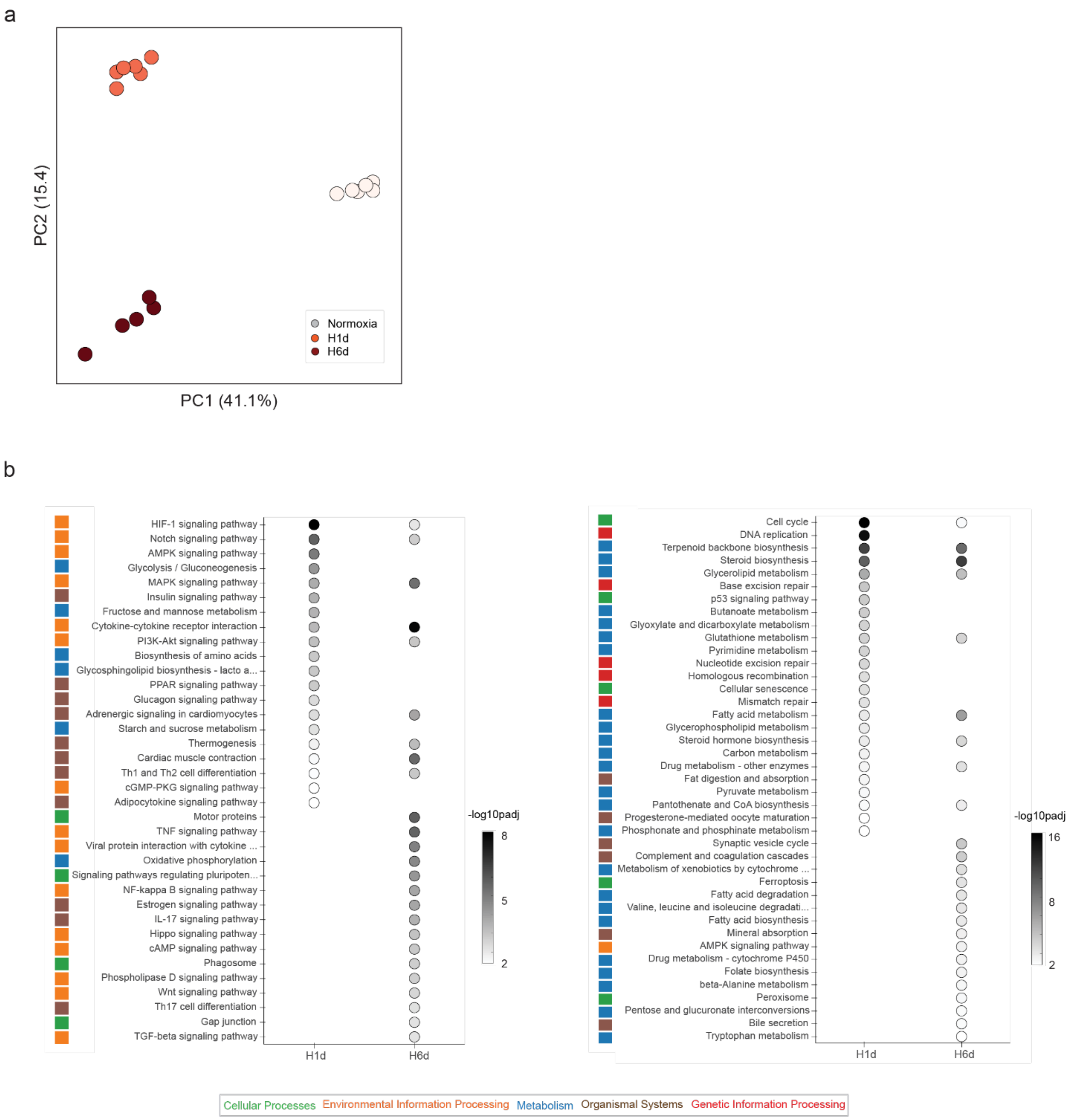

Supplementary Figure 2. Transcriptomic profiles of HepG2 at normoxia, hypoxia 1d, and hypoxia 6d conditions. (**a**) PCA visualization of transcriptomic replicates at each condition. H1d: Hypoxia (1% O_2) 1d. H6d: Hypoxia (1% O_2) 6d. (**b**) Pathway enrichment from (left) up-regulated and (right) down-regulated differentially expressed genes (DE genes) from each hypoxia condition, respectively. DE genes cutoff: $|log_2FC|>1.5$ and *padj*<0.05. Source data are provided as a Source Data file.

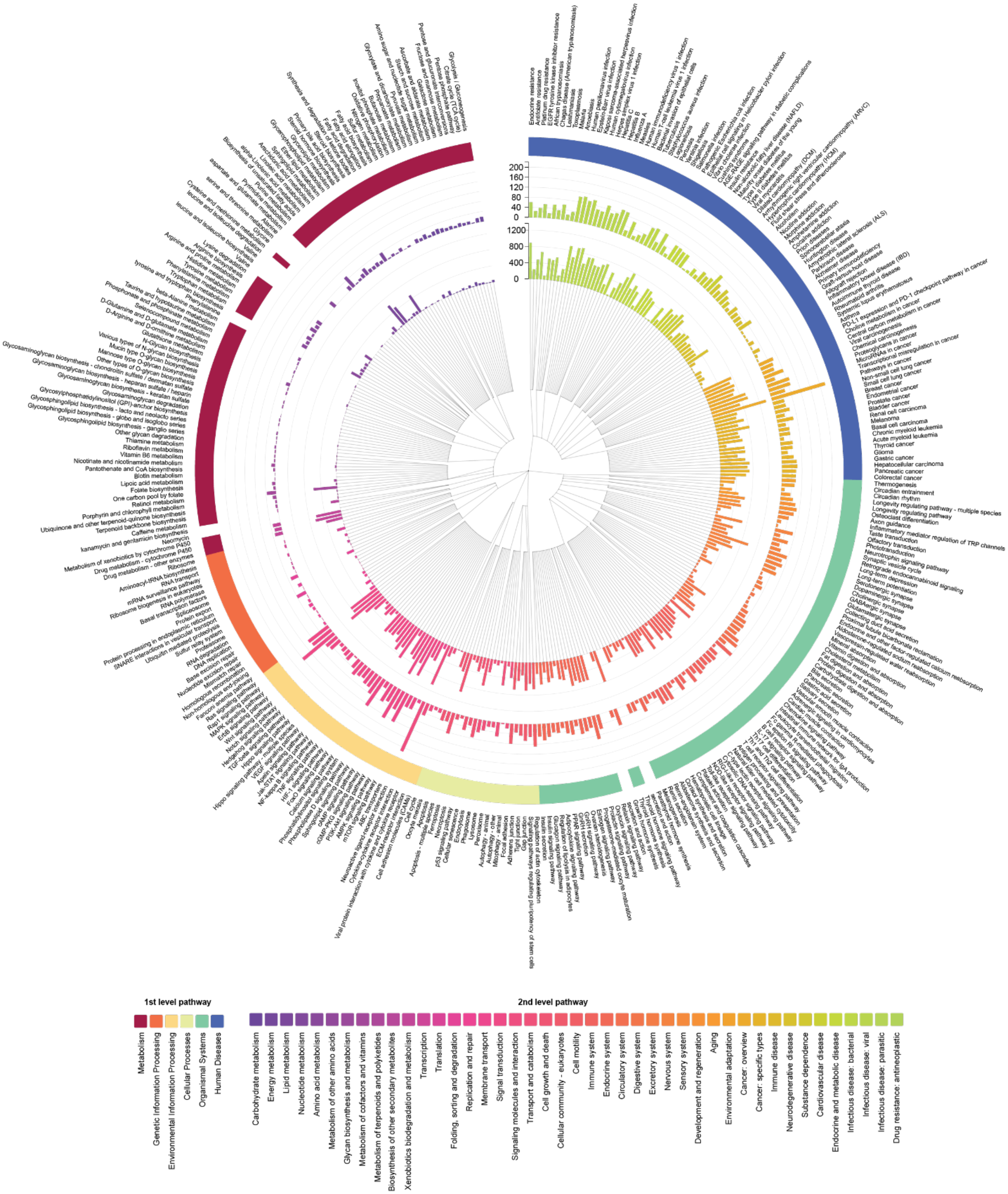

Supplementary Figure 3. Pathway and target coverage of annotated compound library. Coverage of KEGG pathways by the annotated compound library. Inner layer: Number of compounds in each pathway. Middle layer:

Number of covered target proteins in each pathway. Outer layer: KEGG pathway names. Source data are provided as a Source Data file.

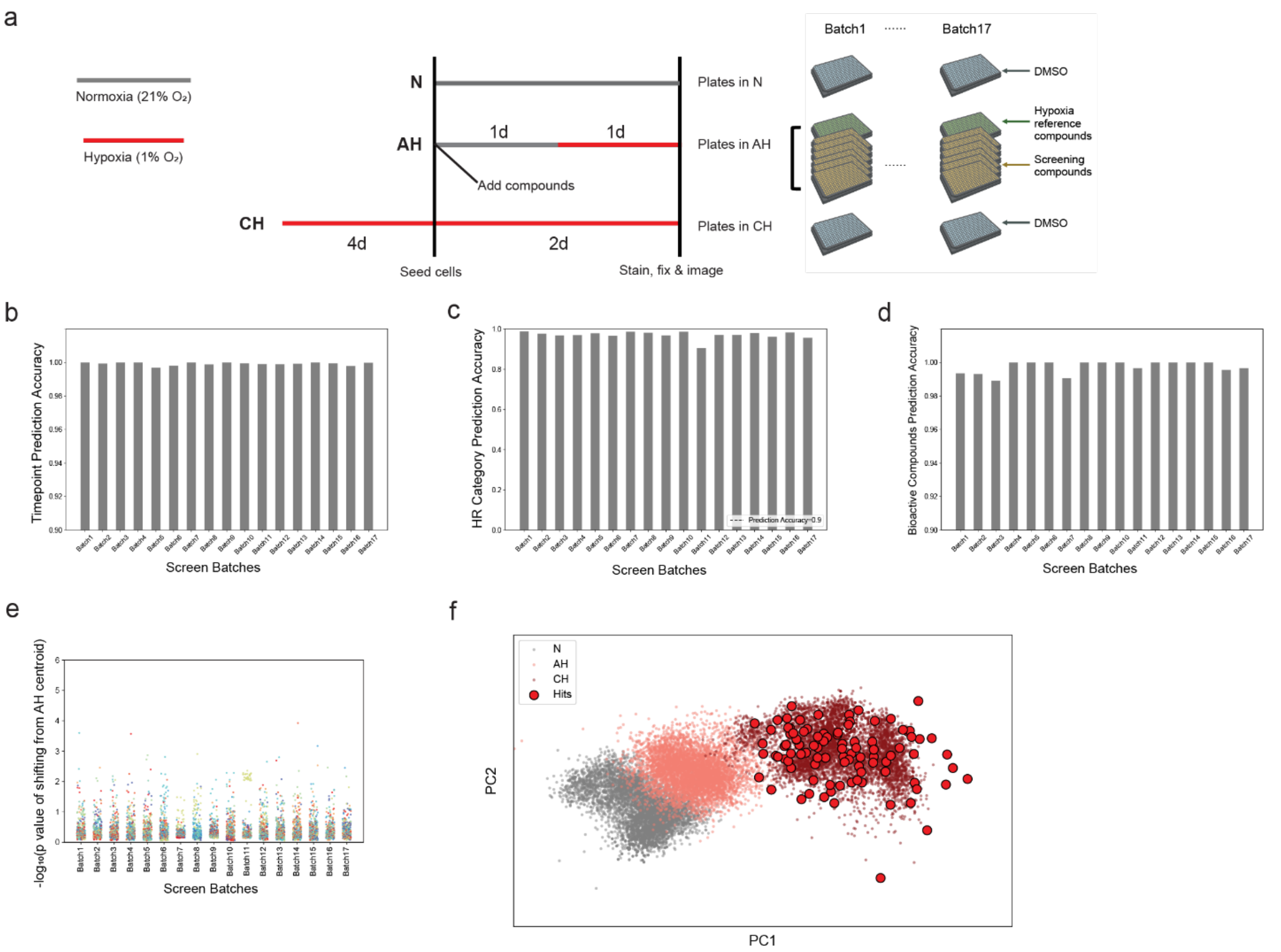

Supplementary Figure 4. AH-to-CH phenopushing screen of the annotated compound library. (a) Experimental timeline and batch design for the screen. N: Normoxia. AH: Acute hypoxia. CH: Chronic hypoxia. The plate icons: Created in BioRender. Hammerlindl, S. (2025) https://BioRender.com/r14u695. **(b-e)** Quality control of phenopushing screen assessed by: **(b)** batch-wise prediction accuracy of hypoxia timepoint for DMSO control wells from N, AH (Hypoxia 1d) and CH (hypoxia 6d), **(c)** batch-wise prediction accuracy of MOA categories of reference compounds in AH (Supplementary Data 2), **(d)** batch-wise prediction accuracy of bioactive control compounds in AH (Supplementary Data 2), and **(e)** bioactivity calculation for DMSO control wells within each batch towards the AH DMSO centroid for that batch (each color represents a testing plate within a batch). **(f)** PCA projection of confirmed hits (red) relative to DMSO controls at different timepoints (N, AH, CH). Source data are provided as a Source Data file.

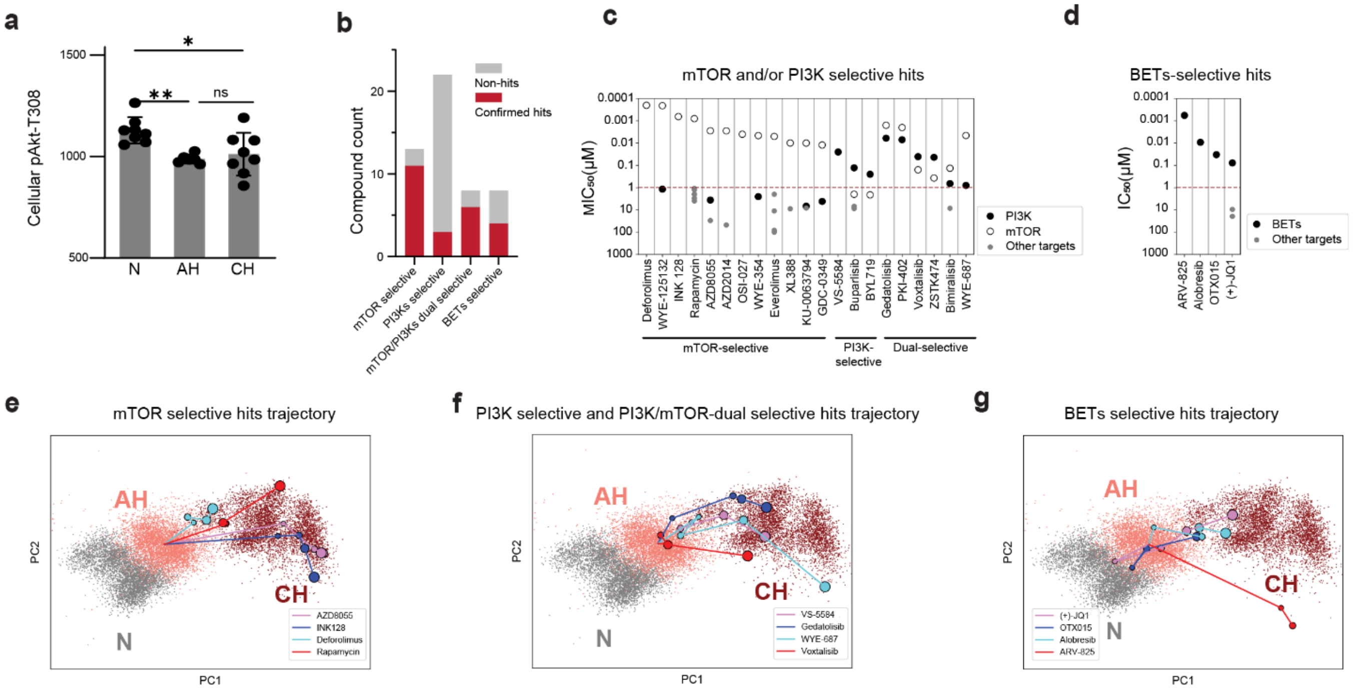

Supplementary Figure 5. Inhibiting mTOR/PI3K or BETs to induce AH-to-CH phenopushing. (a) Quantification of mean cellular pAKT (at S473) in normoxia (N), acute hypoxia (AH, 1d) and chronic hypoxia (CH, 6d) measured by immunofluorescence intensity. Data are shown as mean ± SD from 6~8 biological replicates. One-way ANOVA followed by Tukey's post hoc test: ns: p=0.8304*: p=0.0167, **: p=0.0077. **(b**) Counts of phenopushing hits vs. non-hits in screened compounds that are selective for mTOR, PI3Ks, mTOR/PI3K (dual-selective), and BETs. A compound was considered "selective" for chosen target(s) if only these targets had annotated IC_{50} < 1 μM. **(c, d)** Annotated target activity profiles for phenopushing hits selective for **(c)** mTOR and/or PI3K and **(d)** BETs, based on the IC_{50} (see Methods) for each annotated target**. (e-g)** PCA visualization of dose-dependent phenopushing curves of representative **(e)** mTOR-selective hits, **(f)** PI3K-selective and PI3K/mTOR-dual selective hits, and **(g)** BETs-selective hits. Size of dot reflects concentration (by decreasing size: 10, 2, 0.4, 0.08 μM). For ARV-825, the top 2 doses are not shown due to toxicity. Source data are provided as a Source Data file.

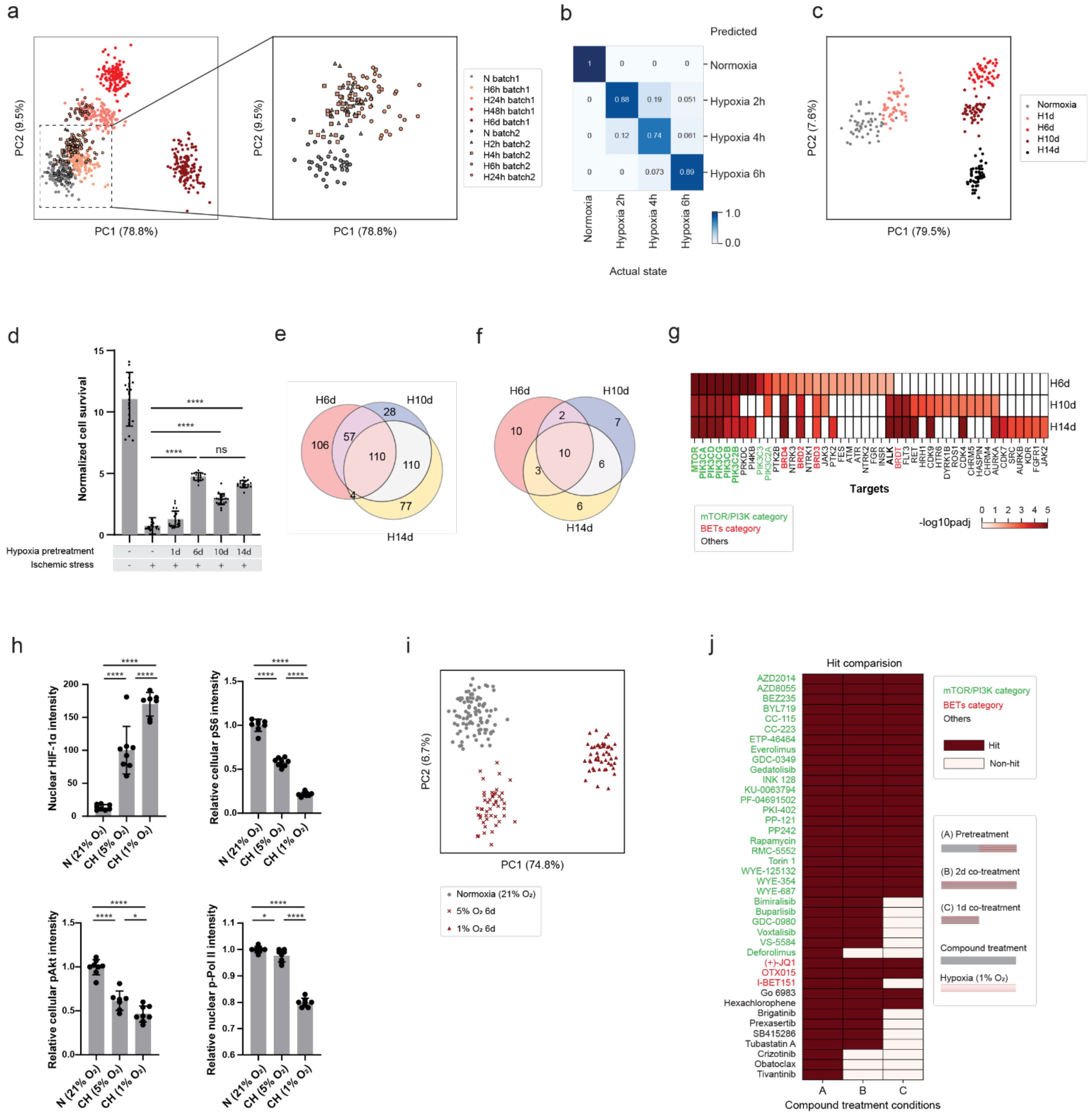

Supplementary Figure 6. Examining other hypoxic states and compound treatment conditions by the phenopushing platform. (a-b) (a) Phenotypic profiling of cellular responses to hypoxia at earlier timepoints (2h, 4h, and 6h) and **(b)** accuracy of distinguishing cellular states by kNN. **(c)** Phenotypic profiling of cellular responses to hypoxia at later timepoints (10d and 14d). **(d)** Normalized cell survival under ischemic stress with different pre-treatment conditions (1d, 6d, 10d and 14d). Data are shown as mean ± SD from at least 10 biological replicates. One-way ANOVA followed by Tukey's post hoc test: ns: p=0.4219, ****: p<0.0001. **(e)** Venn diagram of top-ranking phenopushing compounds across destination states (H6d, H10d or H14d). **(f-g) (f)** Venn diagram and **(g)** list of top enriched targets (by gene name) from overrepresentation analysis of top-ranking phenopushing compounds in (e). Color bar: adjusted p-values for overrepresentation analysis. **(h)** Comparison of molecular responses at the chronic hypoxic state (CH, 6d) under different oxygen levels (5% and 1%) vs. the normoxic state (21% oxygen), based on

immunofluorescence intensity (well-level average of per-cell mean intensity). Top left: nuclear HIF-1α. Top right: cellular pS6 (at S235/S236). Bottom left: cellular pAKT (at S473). Bottom right: nuclear pPol II (at S2). Data are shown as mean ± SD from 7 or 8 biological replicates. One-way ANOVA followed by Tukey's post hoc test: *: p=0.0159 in bottom left plot, p=0.0371 in bottom right plot. ****: p<0.0001. **(i)** Phenotypic profiling of 6d hypoxia response at different oxygen levels (5% and 1%). **(j)** Hit-calling comparison of selected compounds with different compound treatment conditions. Source data are provided as a Source Data file.

Supplementary Table 1. Imaging parameters for staining reagents.

Staining reagent	excitation/emission laser wavelengths [nm]
MitoTracker™ Deep Red FM	615-645/655-760
Hoechst 33342	355-385/430-500
C11-BODIPY	530-560/570-650
BODIPY™ 493/503	460-490/500-550
CellTox™ Green Cytotoxicity Assay	460-490/500-550
Alexa Fluor™ 568 Phalloidin	530-560/570-650
488-conjugated goat anti-mouse IgG	460-490/500-550
488-conjugated goat anti-rabbit IgG	460-490/500-550
647-conjugated goat anti-mouse IgG	615-645/655-760

Supplementary Table 2. Small molecule screening data.

Category	Parameter	Description
Assay	Type of assay	High-content phenotypic screening
	Target	Target-agnostic screening
	Primary measurement	High-dimensional phenotypic profiles extracted from cell images
	Key reagents	Hoechst 33342(Invitrogen, H3570),<br><br>BODIPY™ 493/503 (4,4-Difluoro-1,3,5,7,8-Pentamethyl-4-Bora-3a,4a-Diaza-s-Indacene, Invitrogen, D3922),<br><br>C11-BODIPY (4,4-Difluoro-1,3,5,7,8-Pentamethyl-4-Bora-3a,4a-Diaza-s-Indacene from Image-iT™ Lipid Peroxidation Kit, Invitrogen, C10445),<br><br>MitoTracker™ Deep Red FM (Invitrogen, M22426)
	Assay protocol	HepG2 were seeded 2 days ahead of imaging. Right after plate seeding, compounds were added with a final concentration of 0.1% DMSO. The compound addition was performed by the ECHO 650 liquid handling system (Beckman Coulter, Brea, CA) controlled through a Perkin Elmer EXPLORER G3 WORKSTATION. Screening compounds were tested in duplicate at 2 doses (high dose of 10 µM or 2 µM and a 10-fold dilution).<br><br>After compound addition, cells were treated with indicated oxygen level in Fig 2a. After a total of 48h compound treatment, cells were stained by adding 10 uL of a freshly prepared 8x dye master mix in pre-warmed media (see staining reagents) and incubated at 37˚C for 1h in their corresponding incubators (N plates in normoxic incubator, AH and CH plates in hypoxic incubator). Cells were then fixed by adding 30 µL 16% paraformaldehyde for 30 min at room temperature. Finally, cells were washed three times with 1x HBSS, sealed with adhesive foils for light-protection until imaging. All pipetting steps were performed by MultiFloFX and 405 TS washer (BioTek, Agilent Technologies) controlled through the Perkin Elmer EXPLORER G3 WORKSTATION automation system. Plate handling steps were established using PerkinElmer's plate::works™ (v6.2) software.<br><br>All imaging for the phenotypic profiling of HepG2 cells was performed on the PerkinElmer Operetta CLS System in confocal spinning-disk mode with a 20x water immersion lens (NA1.0, effective resolution 0.66µm). Each well was imaged with 5 fields of view and 4 channels (filters according to wavelength information in Supplementary Table 1) in 3 z-planes. Cell segmentation and the subsequent single-cell feature extraction were performed through the Harmony™ software (v4.9, Perkin-Elmer) based on maximum intensity projections of each field of view.<br><br>Phenotypic profiles were constructed on well-level similar as described in Methods. High-dimensional phenotypic profiles for hypoxic response trajectory section. For the purpose of identifying AH-to-CH phenopushing hits, the phenotypic profiles were calculated using AH DMSO condition as the control. Phenotypic profiles were calculated batch-wise: For a specific feature, the difference in cumulative distribution functions (CDF) between cells in a selected well and cells from control condition (pooled cells from all DMSO-treated wells

		in AH within this batch) were summarized by a Kolmogorov-Smirnov (KS) statistic. KS scores for all features were then concatenated to form a phenotypic profile for the selected well. Subsequent analyses (QC, AH-to-CH phenopushing hit calling) were based on well-level phenotypic profiles
	Additional comments	NA
Library	Library size	6011 compounds
	Library composition	A full list of compounds in the library is presented in Supplementary Data 2.
	Source	Screened compounds included (1) Selleck FDA-approved & Passed Phase I Drug Library (L3800), (2) Selleck Kinase Inhibitor Library (L1200), (3) Selleck Apoptosis Compound Library (L3300), (4) Selleck Epigenetics Compound Library (L1900), (5) Selleck Bioactive Library (L1700), (6) manually curated FDA-approved & Passed Phase I drugs from other vendors, and (7) manually curated bioactive compounds from other vendors.
	Additional comments	NA
Screen	Format	384-well plate.
	Concentration(s) tested	Primary screening: high dose of 10 μM or 2 μM and a 10-fold dilution.<br>Validation screening: 10 μM, 2 μM, 0.4 μM, 0.08 μM, 0.016 μM, and 0.003 μM
	Plate controls	Normoxia, acute hypoxia and chronic hypoxia state (represented by DMSO-treated wells in each condition) plates were included in each batch as controls for screening condition. A compound plate testing a reference library was included in each batch as quality control for screening protocols. Control wells (DMSO wells as negative control, AZD8055 or Fluvastatin treated wells as bioactive control) on each AH plate.
	Reagent/ compound dispensing system	Compounds are dispensed by ECHO 650 liquid handling system.<br>Fixing and Staining reagents are added through Perkin Elmer EXPLORER G3 WORKSTATION and performed by MultiFloFX and 405 TS washer (BioTek, Agilent Technologies).
	Detection instrument and software	Images are collected by PerkinElmer Operetta CLS System in confocal spinning-disk mode with a 20x water immersion lens (NA1.0, effective resolution 0.66μm).<br>Cell segmentation and single-cell feature extraction were performed using the Harmony™ software (v4.9, Perkin-Elmer) based on maximum intensity projections of each field of view.

	Assay validation/QC	Quality control for compound screening was performed on both batch-level and plate-level.<br><br>Batch-level QC. Separability between normoxia, acute hypoxia and chronic hypoxia states (represented by DMSO-treated wells in each condition) as well as the classification accuracy of compounds within a reference library was used as quality control for each batch. (1) Cellular states prediction using phenotypic profiles of DMSO-treated wells was performed on well-level phenotypic profiles by k-Nearest Neighbor (kNN) classifier in the same procedure as described in the Classification of cellular states based on phenotypic profiles section (Supplementary Fig. 4b). (2) Compound category classification for reference compound set (Supplementary Data 2) was performed on well-level phenotypic profiles in the same procedure as cell state classification, except that the phenotypic profiles of selected compound wells were first transformed by linear discriminant analysis with category names as the labels before kNN classification. (Supplementary Fig. 4c)<br><br>Plate-level QC. The plate-level QC was performed by analyzing the phenotypic profiles of control wells (DMSO wells as negative control, AZD8055 or Fluvastatin treated wells as bioactive control) on AH plates. (1) For each batch, compound classification was performed on DMSO and bioactive (AZD8055, Fluvastatin) control wells in all AH plates. (Supplementary Fig. 4d). (2) The Mahalanobis distance of the phenotypic profiles of each DMSO control wells to centroid of the pooled AH DMSO control wells within the same batch were calculated (as indicated later in the section AH-to-CH phenopushing hit-calling for primary screen) and compared for plate-level variability. (Supplementary Fig. 4e)
	Correction factors	Feature selection was performed for batch bias removal. We first calculated PCA embeddings based on combining all well-level phenotypic profiles from all batches. Among the top 5 PCs, we identified the PC dimension that accounted for the most variance. We further checked the feature loading scores for PC2 and identified the top 4 feature types with the strongest batch effects and removed them for subsequent analysis. Computations were performed using Python (v3.9).
	Normalization	NA
	Additional comments	NA
Post-HTS analysis	Hit criteria	Primary screening hit criteria: (1) A tested perturbation is identified as hit perturbation if at least one of the duplicate wells is bioactive (bioactivity p-value $<10^{-6}$) and has $ZScore_i \leq -3$ (where lower $ZScore_i$ is "desirable", i.e., indicates a closer distance towards the chronic hypoxia centroid).Or (2) A tested perturbation is identified as hit perturbation if at least one of the duplicate wells is bioactive (bioactivity p-value $<10^{-6}$) and has θ_i within the top 90% tile of the θ_{CH} population ($\theta_i \leq P_{90}(\theta_{CH})$).<br><br>Validation screening hit criteria: A perturbation (compound + dose) was identified as a hit perturbation if at least 2 out of 3 well replicates meet the hit criteria. The list of confirmed phenopushing hit compounds reflected all compounds with hit perturbations at any dose.
	Hit rate	1.6%

Additional assay(s)	(2) Downstream molecular function test on mTOR and BETs inhibition in HepG2 cells<br>(3) Protection against ischemia in HepG2 cells.<br>(4) Protection against hypoxic stress in iPSC-CM cells.
Confirmation of hit purity and structure	We didn’t include this step since all screening compounds are purchased from vendors with Certificate of Analysis.
Additional comments	NA

Supplementary Table 3. Peak features for iPSC-CMs.

Peak features	
peak_areas_mean	peak_intervals_max
peak_areas_std	peak_intervals_median
peak_areas_min	peak_risetime_mean
peak_areas_max	peak_risetime_std
peak_areas_median	peak_risetime_min
peak_amplitudes_mean	peak_risetime_max
peak_amplitudes_std	peak_risetime_median
peak_amplitudes_min	peak_falltime_mean
peak_amplitudes_max	peak_falltime_std
peak_amplitudes_median	peak_falltime_min
peak_widths_mean	peak_falltime_max
peak_widths_std	peak_falltime_median
peak_widths_min	frequency_mean
peak_widths_max	frequency_std
peak_widths_median	frequency_min
peak_intervals_mean	frequency_max
peak_intervals_std	frequency_median
peak_intervals_min	
