# Supplementary material for: A phenopushing platform to identify compounds that alleviate acute hypoxic stress by fast-tracking cellular adaptation
Source: Nat Commun. 2025 Mar 18;16:2684. doi: 10.1038/s41467-025-57754-1 (PMC11920246; doi:10.1038/s41467-025-57754-1)
Supplement: Supplementary file 2 — Reporting Summary [file 41467_2025_57754_MOESM2_ESM.pdf]

nature portfolio

Corresponding author(s): Steven Altschuler, Lani Wu

Last updated by author(s): Feb 2, 2025

Reporting Summary

Nature Portfolio wishes to improve the reproducibility of the work that we publish. This form provides structure for consistency and transparency in reporting. For further information on Nature Portfolio policies, see our Editorial Policies and the Editorial Policy Checklist.

Statistics

For all statistical analyses, confirm that the following items are present in the figure legend, table legend, main text, or Methods section.

n/a	Confirmed	
[ ]	[x]	The exact sample size (*n*) for each experimental group/condition, given as a discrete number and unit of measurement
[ ]	[x]	A statement on whether measurements were taken from distinct samples or whether the same sample was measured repeatedly
[ ]	[x]	The statistical test(s) used AND whether they are one- or two-sided<br>*Only common tests should be described solely by name; describe more complex techniques in the Methods section.*
[ ]	[x]	A description of all covariates tested
[ ]	[x]	A description of any assumptions or corrections, such as tests of normality and adjustment for multiple comparisons
[ ]	[x]	A full description of the statistical parameters including central tendency (e.g. means) or other basic estimates (e.g. regression coefficient) AND variation (e.g. standard deviation) or associated estimates of uncertainty (e.g. confidence intervals)
[ ]	[x]	For null hypothesis testing, the test statistic (e.g. *F*, *t*, *r*) with confidence intervals, effect sizes, degrees of freedom and *P* value noted<br>*Give P values as exact values whenever suitable.*
[x]	[ ]	For Bayesian analysis, information on the choice of priors and Markov chain Monte Carlo settings
[x]	[ ]	For hierarchical and complex designs, identification of the appropriate level for tests and full reporting of outcomes
[x]	[ ]	Estimates of effect sizes (e.g. Cohen's *d*, Pearson's *r*), indicating how they were calculated

Our web collection on statistics for biologists contains articles on many of the points above.

Software and code

Policy information about availability of computer code

Data collection	Imaging data were collected using the Harmony™ software (v4.9, Perkin-Elmer) based on maximum intensity projections of each field of view. Well-level phenotypic profiles were constructed on well-level to reflect the feature distributions using Python codes (v3.9) accessible on Zenodo at the following link: https://doi.org/10.5281/zenodo.14837176.<br>Beating pattern data of iPSC-derived cardiomyocytes were collected by EVOS M7000 Imaging System. Mean fluorescence intensities for each field of view were extracted using the scikit-image (v1.2.2) module. The signal was baseline-corrected using the Python package BaselineRemoval (v0.1.3).
Data analysis	The code necessary to replicate the results has been made available in Jupyter Notebooks and can be accessed on Zenodo at the following link: https://doi.org/10.5281/zenodo.14837176.

For manuscripts utilizing custom algorithms or software that are central to the research but not yet described in published literature, software must be made available to editors and reviewers. We strongly encourage code deposition in a community repository (e.g. GitHub). See the Nature Portfolio guidelines for submitting code & software for further information.

Data

Policy information about availability of data

All manuscripts must include a data availability statement. This statement should provide the following information, where applicable:

- Accession codes, unique identifiers, or web links for publicly available datasets
- A description of any restrictions on data availability
- For clinical datasets or third party data, please ensure that the statement adheres to our policy

RNA-seq data from GTEx and CCLE FPKM datasets can be accessed from the EMBL-EBI database under the accession numbers E-MTAB-5214 and E-MTAB-2770, respectively.
RNA-seq data generated for this manuscript is deposited in the Gene Expression Omnibus (GEO) repository (GSE283995).
Processed cell line data relevant to each figure has been deposited in CSV format on Zenodo at the following link: https://doi.org/10.5281/zenodo.14837176

Research involving human participants, their data, or biological material

Policy information about studies with human participants or human data. See also policy information about sex, gender (identity/presentation), and sexual orientation and race, ethnicity and racism.

Reporting on sex and gender	This information has not been collected.
Reporting on race, ethnicity, or other socially relevant groupings	This information has not been collected.
Population characteristics	This information has not been collected.
Recruitment	This information has not been collected.
Ethics oversight	This information has not been collected.

Note that full information on the approval of the study protocol must also be provided in the manuscript.

Field-specific reporting

Please select the one below that is the best fit for your research. If you are not sure, read the appropriate sections before making your selection.

☒ Life sciences ☐ Behavioural & social sciences ☐ Ecological, evolutionary & environmental sciences

For a reference copy of the document with all sections, see nature.com/documents/nr-reporting-summary-flat.pdf

Life sciences study design

All studies must disclose on these points even when the disclosure is negative.

Sample size	48~128 biological (well) replicates were used to define the hypoxia response trajectory by phenotypic profiling (Fig. 1b, Supplementary Fig. 6a, 6c and 6i). 6 biological replicates were included in detection of HIF1a accumulation (Fig. 1d). 6 biological replicates were included in detection of metabolism shift (Fig. 1e). At leasey10 biological replicates were included in the detection of the protective effect of chronic hypoxia in ischemic conditions (Fig. 1f, Supplementary Fig. 6d). For the annotated compound library used in the phenopushing screen (Fig. 2), the number of compounds curated (6011) was based on target coverage. 2 well replicates X 2 doses were included for each tested compound in the primary screen. 3 well replicates X 6 doses were included for primary hits in the validation screen. At least 4 biological replicates were included in the study of compound hits' downstream mechanisms (Fig. 3e-g, Supplementary Fig. 5a, Supplementary Fig. 6h) and functional protections (Fig. 4 and 5). All experiments were repeated at least 3 times with similar results.
Data exclusions	We did not exclude any samples in the analysis.
Replication	At least three biological (well) replicates were included in all experiments, except in the primary AH-to-CH phenopushing screening, in which two replicates were included. All findings were successfully repeated in independent experiments.
Randomization	When multiple compounds were tested within one experiment (Figures 2 to 5), compounds were randomized on the plates rather than being grouped by mechanism of action. Within each experiment, plates were stained and imaged in a randomized order.
Blinding	All data collections were performed without knowing the labels of the samples. Within each experiment, data analysis were first performed with only the group labels (negative control or test). The exact metadata, such as the compound name and target information, were added from platemaps after all analysis was completed.

Reporting for specific materials, systems and methods

We require information from authors about some types of materials, experimental systems and methods used in many studies. Here, indicate whether each material, system or method listed is relevant to your study. If you are not sure if a list item applies to your research, read the appropriate section before selecting a response.

Materials & experimental systems

n/a	Involved in the study
☐	☒ Antibodies
☐	☒ Eukaryotic cell lines
☒	☐ Palaeontology and archaeology
☒	☐ Animals and other organisms
☒	☐ Clinical data
☒	☐ Dual use research of concern
☒	☐ Plants

Methods

n/a	Involved in the study
☒	☐ ChIP-seq
☒	☐ Flow cytometry
☒	☐ MRI-based neuroimaging

Antibodies

Antibodies used

1. HIF-1α (Cell Signaling technology, 36169, 1:200).
2. Phospho-S6 Ribosomal Protein (Ser235/236) (Cell Signaling technology, 4856, 1:1600)
3. Phospho-Akt (Ser473) (Cell Signaling technology, 4060, 1:400)
4. RNA polymerase II CTD repeat YSPTSPS (pSer2) (Abcam, ab193468, 1:100).
5. Phospho-Akt (Thr308) (Cell Signaling technology, 13038, 1:400).
6. Sarcomeric alpha Actinin Monoclonal Antibody (EA-53) (Invitrogen, MA1-22863, 1:200).

Validation

1. https://www.cellsignal.com/products/primary-antibodies/hif-1a-d1s7w-xp-rabbit-mab/36169
2. https://www.cellsignal.com/products/primary-antibodies/phospho-s6-ribosomal-protein-ser235-236-2f9-rabbit-mab/4856
3. https://www.cellsignal.com/products/primary-antibodies/phospho-akt-ser473-d9e-xp-rabbit-mab/4060
4. https://www.abcam.com/en-us/products/primary-antibodies/rna-polymerase-ii-ctd-repeat-ysptsps-phospho-s2-antibody-epr18855-ab193468
5. https://www.cellsignal.com/products/primary-antibodies/phospho-akt-thr308-d25e6-xp-rabbit-mab/13038
6. https://www.thermofisher.com/antibody/product/Sarcomeric-alpha-Actinin-Antibody-clone-EA-53-Monoclonal/MA1-22863

Eukaryotic cell lines

Policy information about cell lines and Sex and Gender in Research

Cell line source(s)

HepG2 cells were obtained from the UCSF Cell Culture Core Facility. Human induced pluripotent stem cell (iPSC)-derived cardiomyocytes were generated from a GCaMP6-expressing cell line in the WTC background (obtained from the Gladstone Institute Stem Cell Core facility; this cell line also harbors an inducible dCas9-KRAB cassette and is described in Mandegar et al 2016, Cell Stem Cell, 18, 541–553)

Authentication

HepG2 cells were authenticated by Cell Line Short Tandem Repeat profiling at the University of California, Berkeley Cell Culture Facility in 2024.

Mycoplasma contamination

All cell lines used were tested negative for mycoplasma contamination.

Commonly misidentified lines
(See ICLAC register)

No misidentified lines were used.

Plants

Seed stocks

Report on the source of all seed stocks or other plant material used. If applicable, state the seed stock centre and catalogue number. If plant specimens were collected from the field, describe the collection location, date and sampling procedures.

Novel plant genotypes

Describe the methods by which all novel plant genotypes were produced. This includes those generated by transgenic approaches, gene editing, chemical/radiation-based mutagenesis and hybridization. For transgenic lines, describe the transformation method, the number of independent lines analyzed and the generation upon which experiments were performed. For gene-edited lines, describe the editor used, the endogenous sequence targeted for editing, the targeting guide RNA sequence (if applicable) and how the editor was applied.

Authentication

Describe any authentication procedures for each seed stock used or novel genotype generated. Describe any experiments used to assess the effect of a mutation and, where applicable, how potential secondary effects (e.g. second site T-DNA insertions, mosiacism, off-target gene editing) were examined.
