# Supplementary material for: A phenopushing platform to identify compounds that alleviate acute hypoxic stress by fast-tracking cellular adaptation
Source: Nat Commun. 2025 Mar 18;16:2684. doi: 10.1038/s41467-025-57754-1 (PMC11920246; doi:10.1038/s41467-025-57754-1)
Supplement: Supplementary file 3 — Description of Additional Supplementary Files [file 41467_2025_57754_MOESM3_ESM.pdf]

Title: Supplementary Data 1

Description: Feature list for phenotypic profiles.

Title: Supplementary Data 2

Description: Screening library

Title: Supplementary Data 3

Description: Phenopushing hits

Title: Supplementary Data 4

Description: mTOR, BETs, PI3K screened compounds details
